# New York State Climate Impacts Assessment Chapter 01: Introduction

**DOI:** 10.1111/nyas.15202

**Published:** 2024-12-09

**Authors:** Charlie Goff, Shanika Amarakoon, Deja Curtis, Amanda Stevens

**Affiliations:** ^1^ Eastern Research Group, Inc. Chicago Illinois USA; ^2^ Eastern Research Group, Inc. Portsmouth New Hampshire USA; ^3^ Eastern Research Group, Inc. Arlington Virginia USA; ^4^ New York State Energy Research and Development Authority Albany New York USA

**Keywords:** adaptation, climate change, impacts, New York State, projection, resilience, vulnerability

## Abstract

New York State's climate is changing. Temperatures are rising, precipitation is increasing, sea levels are rising, and extreme weather events are becoming more frequent and severe. Over time, these changes are projected to worsen. The New York State Climate Impacts Assessment provides a science‐based analysis of what to expect from climate change in New York. Working with partners across the state, the team assembled to produce this assessment reviewed the latest science and modeling to project and characterize what New York State's climate is likely to look like in the future and how this will affect natural systems, society, and various sectors of the economy. The assessment will enable decision‐makers at all levels—from individual residents, businesses, and landowners to municipal and state government agencies—to better understand and make informed choices about how to plan and prepare for climate change.

## 1 ASSESSMENT INTRODUCTION

New York State's climate is changing. Temperatures are rising, precipitation is increasing, sea levels are rising, and extreme weather events are becoming more frequent and severe. Over time, these changes are projected to worsen. The New York State Climate Impacts Assessment provides a science‐based analysis of what to expect from climate change in New York.

Working with partners across the state, the team assembled to produce this assessment reviewed the latest science and modeling to project and characterize what New York State's climate is likely to look like in the future and how this will affect natural systems, society, and various sectors of the economy. The assessment will enable decision‐makers at all levels—from individual residents, businesses, and landowners to municipal and state government agencies—to better understand and make informed choices about how to plan and prepare for climate change.

The assessment builds on New York's first statewide climate change impacts assessment (ClimAID), published in 2011^1^ and updated in 2014.^2^ Since 2014, scientific understanding of climate change has continued to advance, and climate models have evolved significantly. The assessment team incorporated those advances—as well as input from a greater number of stakeholders, decision‐makers, and potential users—to ensure that the information in the assessment is relevant, topical, and accessible to the state's diverse communities and interests.

The peer‐reviewed technical chapters include

- Up‐to‐date projections of future climate conditions in New York State.
- Sector‐specific assessments of climate impacts based on thorough literature reviews.
- Adaptation strategies and case studies.
- Links and references to primary sources for full transparency.

In addition to the technical chapters, the assessment team produced a variety of communication and outreach materials to present information from the assessment.

### 1.1 Why is an impact assessment important?

This assessment focuses primarily on the **impacts of climate change** in New York State. A multitude of climate impacts pose risks to every one of the state's economic sectors, industries, natural systems, communities, and regions. For example, the agricultural growing season is changing; coastal and inland flooding is happening more often; populations of plants and wildlife are shifting; and the state's energy and transportation systems are becoming increasingly stressed. In addition, although all communities feel the impacts of climate change, some people—particularly Indigenous Peoples and members of historically marginalized racial and ethnic groups—face greater burdens and disproportionate impacts. Even if local and global efforts succeed at reducing the greenhouse gas emissions that are the primary cause of climate change, New York State's climate has already changed, impacts are already being felt, and some amount of further climate change has already been set in motion. Thus, it is critical to understand and prepare for the impacts of climate change.

Although climate impacts are increasing, New York State has tremendous adaptive capacity to respond through its people, processes, and technologies, as examples throughout this assessment's technical chapters show. The better New Yorkers understand the impacts of climate change, the better they can prepare. Furthering understanding of future climate impacts can help inform action and adaptability at every level to ensure the health, safety, and resilience of the state's communities, especially those that disproportionately experience the adverse effects of climate change.

### 1.2 Scope of the assessment

This assessment presents and evaluates historical observations and future projections of

- **Climate hazards**—the physical manifestations of climate change, such as changes in temperature, precipitation, extreme weather events, and sea level.
- **Climate impacts**—how climate hazards affect ecosystems, natural resources, infrastructure (the built environment), human health and well‐being, communities, and the economy. This cross‐sector perspective helps reveal how impacts can lead to other impacts, which can lead to still more impacts. This assessment seeks to consider such impacts, acknowledging their interdependencies and cascading connections.

The assessment provides an overview of climate hazards and impacts projected to affect New York State. It also assesses vulnerabilities to these hazards and impacts, with an eye toward acknowledging inequities and advancing just solutions that address them.

In addition to detailing the impacts the state is experiencing and projected to experience, this assessment presents information about **adaptation and resilience** (refer to Box 1 for definitions of these and other key terms). It does not aim to provide exhaustive coverage of these topics; for example, it does not list every possible adaptation or resilience strategy. It also does not aim to recommend specific strategies, as doing so would arguably require detailed case‐specific cost–benefit analysis that is beyond the scope of this effort. What this assessment does do, however, is highlight examples of key strategies or types of strategies that are already in use or could be used, share published knowledge on the efficacy of key strategies where it exists, and point readers to additional resources that can further support decision‐making.

The assessment does *not* focus on the sources of greenhouse gas emissions and strategies for reducing them (i.e., greenhouse gas mitigation). Such topics are worthy of their own volume of work—and indeed, they are covered by separate ongoing efforts in New York. Readers can learn more about the state's critical efforts to track and reduce greenhouse gas emissions through the New York State Department of Environmental Conservation (NYSDEC) and the statewide Climate Act web portal.

Ultimately, this effort seeks to clearly demonstrate the necessity of taking action now to adapt to future climate conditions.

BOX 1 **Key terms**

Several key terms that appear throughout the assessment are defined below. Readers can find a glossary with additional terms used in the assessment at https://nysclimateimpacts.org/resources/glossary.

**Projection**: The simulated response of the climate system to a scenario of future emissions or concentrations of greenhouse gases and aerosols generally derived using climate models. Climate projections depend on the emissions, concentration, or radiative forcing scenario used, which, in turn, is based on assumptions concerning, for example, future socioeconomic and technological developments that may or may not be realized.^3^

**Vulnerability**: The degree to which physical, biological, and socioeconomic systems are susceptible to and unable to cope with adverse impacts of climate change.^4^

**Adaptation**: Adjustment in natural or human systems to a new or changing environment that seizes beneficial opportunities or moderates negative effects. Alternatively, it is the process by which a system moves toward resilience. Practitioners increasingly recognize that adaptation has many dimensions, and they use tools such as the Awareness, Coping, Hazard Mitigation, and Adaptation (ACMA) framework^5^ to consider multiple parallel opportunities to assess climate change impacts and readiness across complex systems:

- **Awareness** refers to efforts to improve communication of climate impacts so community members understand the changing climate and consider their individual or family exposure to those changes. Examples include heat‐health risk awareness campaigns or Know Your Zone campaigns for evacuation.
- **Coping** refers to public resources that are available to community members, in addition to their own family resources, to deal with direct and indirect climate impacts. Examples include having access to alternative power, such as a generator, in case of power outages or having access to a cooling shelter in times of extreme heat. Coping strategies offer short‐term respite from climate‐induced risks.
- **Hazard mitigation** refers to tactics for reducing the direct and indirect impacts of climate change or mitigating its effects. Examples include weatherization programs that enable community members to manage increased temperature while sheltering safely in their homes; the use of home‐raising to allow floodwater to move through a parcel; or the use of elevation‐aware parking to remove vehicles from floodwater conveyance areas.
- **Adaptation** (within the ACMA framework) refers to larger scale changes designed to address the mid‐ and longer term impacts of climate change. Examples include large‐scale engineering of storm surge barriers, elevation‐based zoning to reduce the number of homes in the floodplains, or legislative changes such as the Community Risk and Resiliency Act.

As used elsewhere in this assessment, the umbrella term “adaptation” encompasses all four of these components.

**Resilience**: A capability to anticipate, prepare for, respond to, recover from, and adapt to significant multi‐hazard threats with minimum damage to social well‐being, public health, the economy, and the environment.^4^

**Climate equity**: The principle that all residents should have a fair and just opportunity to live, learn, work, and play in a safe, healthy, resilient, and sustainable environment, even as the climate changes.^6^

**Climate justice**: The promotion of individual and collective capacity to prepare for, respond to, and recover from climate events, as well as fair treatment, meaningful involvement, and absence of discrimination in the creation of policies, programs, and projects that address both the disparate impacts of climate change and the transition to a net‐zero carbon emissions economy.^7^

### 1.3 Sectors

The assessment addresses climate change impacts across all of New York State's communities, natural systems, and economic sectors and industries. The assessment is organized into nine technical chapters: One focuses on physical climate observations and projections, whereas the other eight each focus on climate change impacts within a particular sector (refer to Figure 1-1). As described in Section 2, the chapters were developed by teams of expert authors and other contributors.

**FIGURE 1-1:**
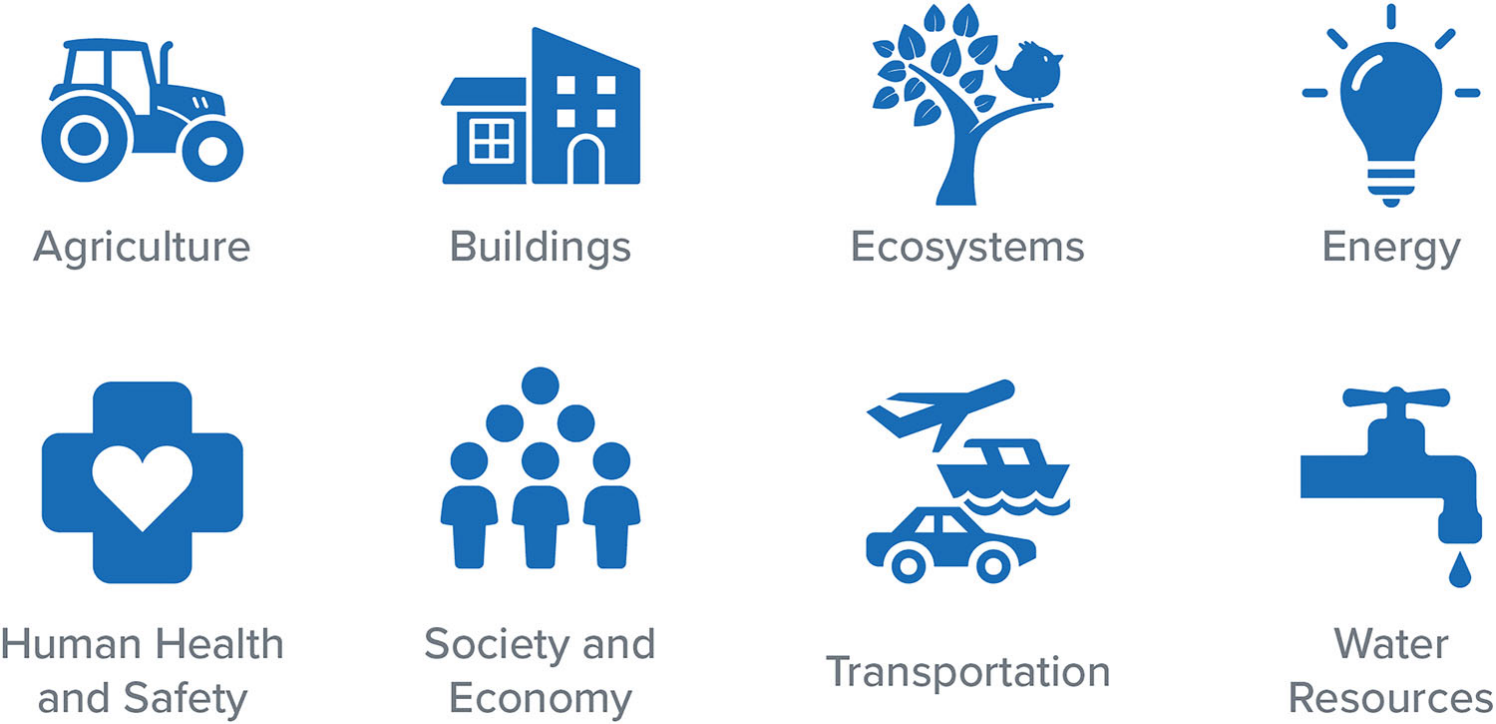
Sectors covered in the assessment.

Each sector chapter begins with a presentation of the authors’ key findings regarding climate impacts in the sector. Chapters then introduce sector‐specific scope and context; this section includes a sector description, a summary of key climate hazards, a summary of other critical factors that intersect with climate hazards, an overview of equity and climate justice considerations, and highlights of the key climate impacts, perspectives, issues, and equity considerations relevant to Indigenous Peoples in New York. Subsequent sections present observed and projected impacts of climate change in the sector, vulnerable populations and systems that face particular risks from climate change, and strategies for adapting and building resilience to the impacts discussed.

Each sector chapter concludes with “traceable accounts” that connect the dots between the evidence presented in the chapter and the key findings presented. This section describes the evidence base and uncertainties related to each key finding as well as summarizes the authors’ level of confidence in each finding. The technical chapters also link to a suite of case studies that illustrate climate impacts and adaptation/resilience responses in specific communities across New York State. Many of these case studies help to put a human face on climate change, showing the very real ways in which climate change affects New Yorkers’ lives and livelihoods, while also sharing inspiring stories of how the innovative, resilient people of New York have begun to take action.

### 1.4 Regions

The assessment addresses climate impacts across all of New York State. New York is a large state with diverse geographical features and varied climate conditions, related to factors including elevation and proximity to water bodies such as the Great Lakes and the Atlantic Ocean. As a result, the impacts from climate change and the feasibility of some adaptation strategies can vary significantly in different areas of the state. To provide a standardized framework for the climate modeling and for analyzing regional differences, this assessment uses a set of 12 regions, as shown in Figure 1-2. These regions were selected to balance a variety of considerations and commonalities, including jurisdictional boundaries, geographic features, historical climatology, and climate data availability (each region having at least one weather station with suitable long‐term data). The regions align more closely with the US Climate Divisions than did the regions used in previous assessments.

**FIGURE 1-2:**
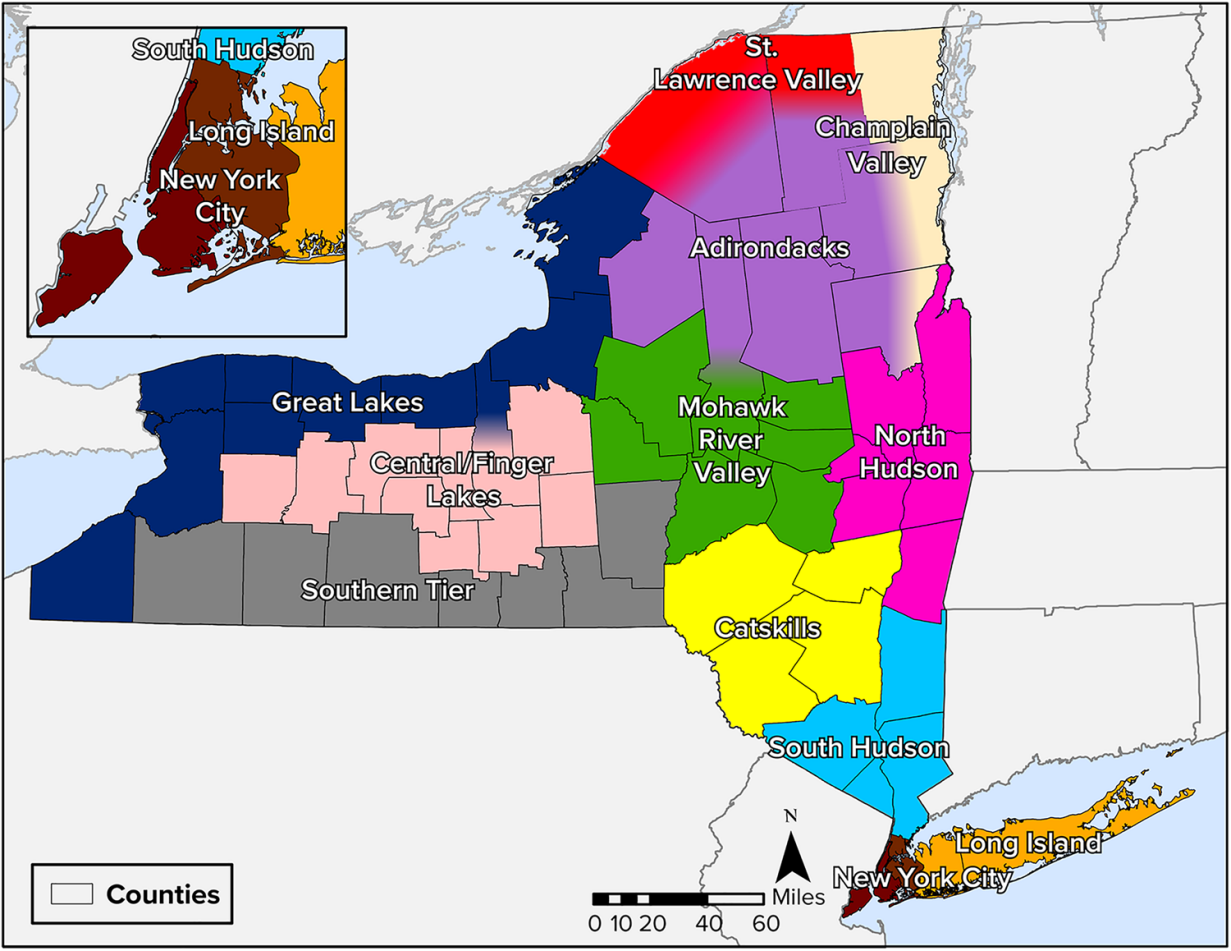
Regions used in the New York State Climate Impacts Assessment.

Although the region boundaries generally follow county borders, several counties span multiple regions. The intent is to highlight that some counties encompass diverse geographies and experience a range of climate hazards and therefore may experience varied climate change impacts. For example, in Essex County, low‐elevation areas in the Champlain Valley may experience a different climate than higher elevation areas deeper within the Adirondack Mountains.

Chapter 2, New York State's Changing Climate, presents data on observed and projected weather and climate trends across the state's regions. Each sector chapter discusses regional factors, unique impacts, and adaptation strategies, as appropriate.

### 1.5 Cross‐cutting perspectives

In addition to the eight sectors identified above, the assessment addresses two topic areas that apply to all sectors: (1) equity and underserved communities and (2) municipal government concerns.

Equity and justice considerations are built into the framework of the assessment, so that its findings, data, and information are accessible to, responsive to, and actionable for all communities and provide decision‐makers with knowledge to better address inequities. Climate change will add to the many stresses that overburdened, low‐ to moderate‐income communities already face. In certain areas, both rural and urban, infrastructure may be older or poorly designed, leading to challenges for communities seeking to adapt to the effects of climate change. Recognizing that climate action needs to be effective for all, each chapter addresses how impacts vary for different communities and population groups, how some impacts are worsened by persistent historical injustices and other existing inequities, and how certain adaptation and resilience measures can help to address these disproportionate burdens. Section 4.5 of this chapter provides more information about the diverse communities that make up the fabric of New York State and how equity and justice have been woven throughout the assessment.

The assessment recognizes the vital role that New York State's county, city, town, and village governments play in responding to and preparing for climate change. These municipal governments often find themselves on the front line in responding to climate‐related events or planning proactively for future events. Examples include local emergency responders handling a disaster such as a flood, local health departments and others operating cooling shelters and early warning systems for residents vulnerable to extreme heat, planners addressing land use challenges and opportunities, local decision‐makers adopting stretch building codes, municipal water and wastewater/stormwater utilities building resilience against water supply and precipitation challenges, and public works departments designing and building resilient roads and other infrastructure. Municipal government agencies bear much responsibility for managing the local impacts of global climate change, yet they often must do so with limited financial resources and staff capacity, especially compared with state and federal agencies. Mindful of these roles and challenges, many of the assessment chapters discuss climate impacts that affect local government operations, as well as adaptation measures that local agencies and leaders can take to build more resilient communities.

### 1.6 How this assessment relates to other climate actions and policies in New York State

New York State's Community Risk and Resiliency Act, signed in 2014, required certain planning, permitting, and funding processes statewide to incorporate climate change considerations. For example, applicants for certain permitting and funding programs must consider the impacts of extreme weather, sea level rise, storm surge, and flooding. The law also called for development of model climate change adaptation zoning laws and a standard set of sea level rise projections. This assessment provides updated climate information that programs, planners, and others can use to comply with the provisions of the Community Risk and Resiliency Act.

On July 18, 2019, the Climate Leadership and Community Protection Act (Climate Act) was signed into law. New York State's Climate Act is among the most ambitious climate laws in the nation and requires aggressive economy‐wide reductions in New York's greenhouse gas emissions. The law created a Climate Action Council charged with developing a scoping plan to meet these targets and place New York on a path toward carbon neutrality. By their nature, both the Climate Act and the Climate Action Council focus largely—although not entirely—on greenhouse gas reduction.

In contrast to the Climate Act, this climate assessment is not developing or recommending policy, nor is it focused on how to reduce emissions. Rather, the focus is on how the climate is changing (projections), how those changes will affect New York (impacts), and how New Yorkers can prepare for some impacts that may be uncertain or unavoidable (adaptation and resilience). The assessment provides the science and information that will allow decision‐makers at all levels—whether local municipalities, state agencies, individual businesses, or landowners—to make informed choices about the future. The information in the assessment may form the scientific foundation for future recommendations or iterations of the Climate Act or other New York State policies and initiatives.

## 2 ASSESSMENT TEAM

In a state as diverse as New York, it is crucial that an assessment of this type be informed by a wide range of voices. Following an open call for nominations, members of the assessment team were selected based on their experience, expertise, and insights into the many complex ways that climate change is impacting the state. The assessment team members represent a diversity of perspectives. They come from a variety of organizational structures (academic, nonprofit, municipal, etc.); from all geographic regions of New York State; from urban, suburban, rural, and Tribal communities; and from numerous economic sectors. They represent a wide range of New York State's racial, ethnic, cultural, gender, and socioeconomic identities.

Several teams worked in concert to develop the assessment:

- Technical Workgroups (TWGs)
- Sector Advisors
- A Steering Committee
- The New York State Energy Research and Development Authority (NYSERDA) and its contractors
- The Columbia University climate projections team

### 2.1 Technical workgroups and sector advisors

#### 2.1.1 Technical workgroups

Eight TWGs, representing each of the eight sectors, conducted much of the day‐to‐day work of the assessment, including searching for and critically reviewing the best available scientific literature, incorporating new projections, synthesizing data and information, and developing this technical report. Figure 1-3 illustrates the TWGs’ structure.

**FIGURE 1-3:**
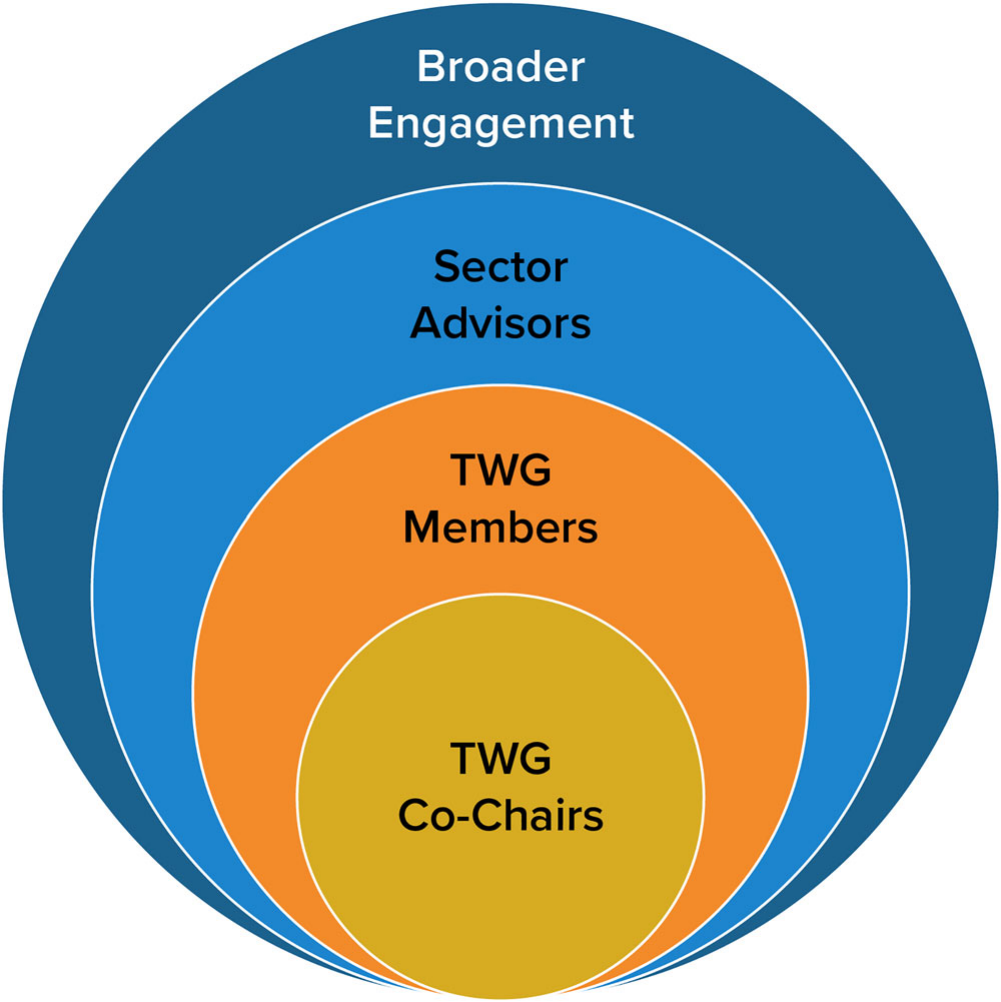
Technical Workgroup (TWG) structure.

Each workgroup consisted of 8–10 TWG members, who compiled and analyzed technical research, developed and wrote specific sections of their sector's chapter, reviewed and revised chapter drafts, gathered information from Sector Advisors, and participated in meetings and workshops. TWG cochairs served as lead authors/convening lead authors of their sector chapter, helped to select TWG members and Sector Advisors (refer to Section 2.1.2), and provided overall leadership for their TWG's activities.

Three subgroups were also established to help inform development of the assessment across sectors: an equity and justice subgroup, an Indigenous perspectives subgroup, and a subgroup focused on developing the assessment glossary. Members of these subgroups included volunteers from across the eight TWGs.

#### 2.1.2 Sector advisors

Each TWG was supported by a diverse group of approximately 20 Sector Advisors—representatives from communities and constituencies that are affected by the impacts of climate change in their sector or active in addressing these impacts. The Sector Advisors contributed expertise, lived experience, and insights to ensure that the assessment captures the impacts that matter most, that the process was driven by the needs of people who will actually use the information contained in the assessment, and that the results are accessible and actionable. As needed, TWGs conducted broader engagement with additional stakeholders to help inform their sectors.

Sector Advisors participated in several assessment workshops, participated in additional topic‐specific or cross‐sector conversations, and provided input and feedback on assessment materials.

### 2.2 Steering committee

The whole assessment was guided by a Steering Committee consisting of climate scientists, assessment experts, and representatives from nonprofit organizations and state and municipal government agencies. This diverse group provided overarching expertise and guidance to the technical experts developing the assessment. Additional detailed support came from a team of Assessment Design Advisors, made up of a subset of Steering Committee members working in collaboration with a small team of external consultants with expertise in developing state, national, and international climate assessments.

### 2.3 NYSERDA's role and contractor support

NYSERDA sponsored the assessment, providing project vision and leadership, management and oversight, review of all assessment products, funding for all assessment activities, and coordination among contractors. NYSERDA was supported by two contractors, Eastern Research Group (ERG) and Consensus Building Institute (CBI).

- **ERG** served as the overall assessment coordinator. Working closely with NYSERDA, ERG helped identify TWG members and Sector Advisors, set project schedules and deliverables, provided collaboration platforms to facilitate TWG activities, and provided supplementary research, writing, editing, and design support for the final assessment chapters and web and outreach content. ERG team members also served as sector liaisons, providing logistical, administrative, and technical support to each of the TWGs.
- **CBI** served as the assessment facilitation coordinator, designing and facilitating key meetings, coordinating support for Sector Advisors, and helping to resolve conflicts and address challenges.

### 2.4 Climate projections team

A team from Columbia University used historical data, climate modeling, and literature review to understand how physical variables like temperature, rainfall, heat waves, extreme precipitation, and sea level are likely to change between now and 2100 (or 2150, in the case of sea level). The team used the best available modeling tools and data to focus on impacts within New York State's individual counties and regions, ensuring that the assessment reflects the current state of the science. They and ERG also reviewed additional projections and historical observations for context. Section 3.3 presents more information about the climate modeling methods and sources.

The climate projections team was advised by a group of climate scientists from the National Oceanic and Atmospheric Administration and from academic institutions throughout the Northeast.

## 3 METHODS AND SOURCES

### 3.1 Literature review, key findings, and traceable accounts

To conduct the assessment, each TWG gathered evidence on the anticipated impacts from climate change through a systematic literature review. Each TWG then weighed the evidence collected and developed key findings and traceable accounts. Refer to Section 3.2 for the types of sources and the data source standards used in the assessment.

**Key findings** represent each TWG's strategic takeaways from its assessment work. Key findings attempt to relay:

- The “What?” (current challenges, circumstances, facts, observations, and projections, as well as an indication of level of confidence).
- The “So what?” (implications, meanings, and inferences from integrating multiple lines of evidence, including underlying assumptions).
- The “Now what?” (potential actions, trade‐offs, resources needed to address the challenges, and knowledge gaps).

Key findings focus on impacts, responses, and solutions. Each TWG also included information in its key findings about equity considerations, including disproportionate impacts and vulnerabilities and associated solutions.

**Traceable accounts** examine each key finding in depth. The traceable accounts section gives TWGs the opportunity to present their expert judgment of the body of evidence for each key finding, as well as their level of confidence in the finding. Each traceable account examines:

- The evidence that supports each key finding.
- The quality of the evidence (e.g., gaps, data, types of analyses, and state of theory).
- The ranges of estimates or interpretations in the literature.
- Assumptions and their implications for what was or was not considered.
- The level of agreement in the literature.
- Key uncertainties.
- The TWG's level of confidence in the key finding (refer to Box 2).

For projected impacts, the traceable accounts specify the climate scenarios used and how the impacts vary among the scenarios considered.

BOX 2 **Confidence level**

**Very high**

Strong evidence (established theory, multiple sources, consistent results, well‐documented and accepted methods, etc.), high consensus.

**High**

Moderate evidence (several sources, some consistency, methods vary and/or documentation limited, etc.), medium consensus.

**Medium**

Suggestive evidence (a few sources, limited consistency, models incomplete, methods emerging, etc.), competing schools of thought.

**Low**

Inconclusive evidence (limited sources, extrapolations, inconsistent findings, poor documentation and/or methods not tested, etc.), disagreement or lack of opinions among experts.

*Source: US Global Change Research Program, Fourth National Climate Assessment*.^8^

### 3.2 Types of sources and data source standards used in the assessment

#### 3.2.1 Types of sources

The goal of this assessment is to capture the best available information about observed and projected impacts of climate change in New York State. The assessment team accessed and reviewed many types of sources, including:

- Peer‐reviewed literature.
- “Gray literature,” which is information produced outside of formal publishing and distribution channels (e.g., government or private‐sector reports, working papers, and white papers).
- Data generated as part of the assessment, following peer‐reviewed methods (e.g., projections developed by the Columbia University team).
- Traditional knowledge (e.g., Indigenous ecological knowledge).
- Case studies, news media, and firsthand accounts (e.g., interviews).

As this list demonstrates, the assessment was not restricted to scientific, peer‐reviewed journal articles. Some types of climate impact information are not contained in peer‐reviewed resources, as such resources have not always incorporated Indigenous and other historically marginalized voices or the experiences of individuals who observe and respond to climate impacts directly. Therefore, the assessment team sought to capture these types of information through firsthand accounts from Sector Advisors and other New Yorkers with direct lived experience (e.g., interviews with farmers). At the same time, an important part of each TWG's role was to assess the relative quality of various inputs. For topics where rigorous, peer‐reviewed sources are available, TWGs relied heavily on those peer‐reviewed sources and used other types of information in a complementary or supplementary capacity.

#### 3.2.2 Public access

To support the assessment goals of transparency and credibility, every source used in the assessment is citable and publicly accessible. Sources not publicly accessible, such as proprietary or otherwise confidential reports, were not used. This policy ensured that the assessment is built on information that is available for inspection and review by any user seeking to corroborate the assessment team's conclusions or learn more about an underlying study's methods and conclusions. Types of publicly accessible sources include:

- Information posted publicly online.
- Resources that can be obtained through reasonable effort, such as by using a library subscription or purchasing access from a publisher. (TWGs were allowed to cite journal articles located behind a publisher's paywall; articles did not need to be open‐access.)
- Traditional or personal knowledge that interested parties can learn more about through online resources or by contacting an interviewee listed in a citation.

#### 3.2.3 Geographic scope

The TWGs placed special emphasis on locating information that characterizes impacts specific to New York State or portions of the state, as this is a primary goal of the assessment. However, in some instances, they incorporated information from outside the state where it filled a gap or provided additional relevant evidence, context, or scientific rigor. In some cases, the TWGs found value in supplementing New York–specific data with information at a regional scale (e.g., the Northeast), information from nearby states or cities, or information from elsewhere that may be transferable to New York State.

#### 3.2.4 Temporal coverage

Another primary goal of the assessment is to be current. Each TWG cited any source that it felt is appropriate, relevant, and accurate, regardless of age. The 2011 ClimAID assessment provided a useful starting point for most sectors; and in some cases where a TWG determined that the ClimAID sources still represent the best available science, this assessment presents similar conclusions based on some of the same sources. However, most of the evidence in the current assessment comes from information published since the 2011 ClimAID assessment. Many of the sources reflect information available as of mid‐2022, which was the initial cutoff for inclusion in the assessment. However, the authors subsequently incorporated newer sources where needed to fill gaps or address comments from external reviewers. The most recent sources included in the assessment are sources published in fall 2023.

### 3.3 Overview of physical climate modeling methods and sources

The Columbia University team developed updated climate science projections for New York State using global climate models that simulate how physical variables like temperature, rainfall, heat waves, and sea level are likely to change between now and 2100 (or 2150 for sea level). The team also relied on literature review, given inherent limitations in climate models. Columbia developed similar projections for the original ClimAID report in 2011 and the update in 2014. Columbia updated these projections to use the newest climate models—part of a suite of models called CMIP6.^9^ The CMIP6 models were recently used in the Intergovernmental Panel on Climate Change's Sixth Assessment Report and the US Global Change Research Program's Fifth National Climate Assessment. They are widely accepted by climate scientists as the most up‐to‐date tools available to simulate the complex connections between greenhouse gases in the atmosphere, Earth's average temperature, and the resulting changes in weather and climate patterns, the oceans, and more. Columbia applied these models to New York State, generating specific projections for each region of the state and comparing them with historical climate data.

The projections methodology report provides additional information about the methods behind Columbia's climate projections, including key uncertainties, sources of baseline data, and a comparison with projections used in New York's 2011 and 2014 ClimAID assessments.^10^ The projections themselves are discussed more fully in New York State's Changing Climate.

## 4 CLIMATE IMPACTS: THE NEW YORK STATE CONTEXT

This section explores New York State's geography, government structure, economy, and demographics, while also presenting historical context on the state's socially vulnerable communities. This information provides a backdrop for understanding the different ways in which climate change will affect New York's regions, economic sectors, and population groups—including disproportionate impacts on vulnerable populations, as each technical chapter explores further.

### 4.1 Geography and key features

Despite being one of the country's most populous states, New York is largely rural, with forests and agricultural parcels covering the majority of the state. As illustrated in Figure 1-4 and summarized in Table 1-1, forests cover 55% of the state, and agricultural land (defined as land used to cultivate crops as well as land used for hay production and pasture)^11^ covers approximately 21%. Approximately 11% of land in the state is categorized as developed.

**FIGURE 1-4:**
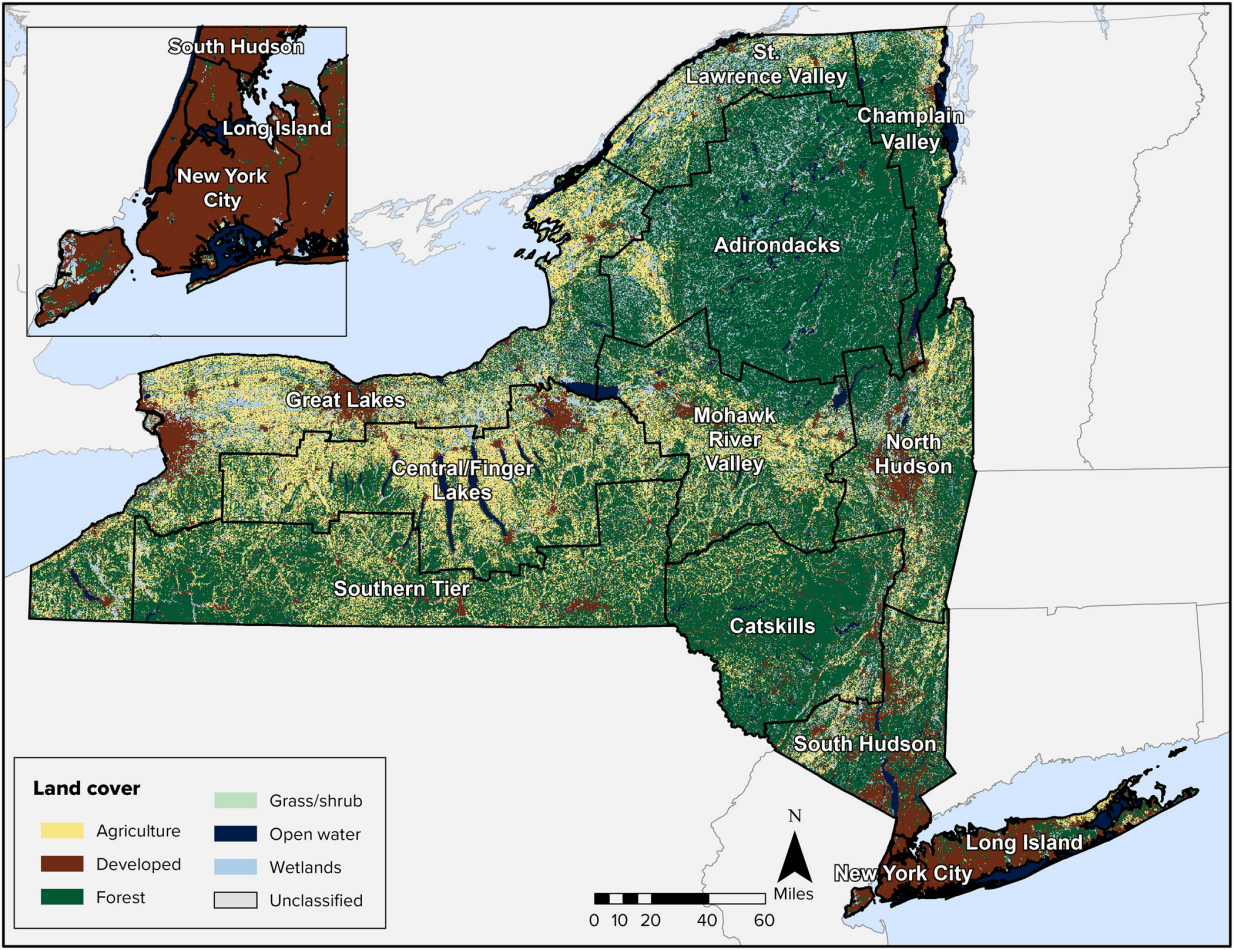
Major land cover types in New York State. *Source*: Data from Dewitz and US Geological Survey (2021).^12^

**TABLE 1-1:** Percent land cover for New York State, the 12 climate assessment regions, and Tribal lands within the state.

Land cover type	Statewide	Tribal lands^a^	Adirondacks	Catskills	Central/Finger Lakes	Champlain Valley	Great Lakes	Long Island	Mohawk River Valley	New York City	North Hudson	South Hudson	Southern Tier	St. Lawrence Valley
Forest	55.0	53.8	77.9	76.6	36.9	67.6	34.1	21.3	53.9	4.0	55.2	49.7	64.2	46.6
Agriculture	20.8	10.6	2.8	9.1	41.1	10.6	34.4	4.7	25.9	0.2	19.3	10.9	23.8	22.5
Developed	11.1	8.2	2.2	7.1	10.1	6.5	14.0	64.0	7.7	89.2	12.9	25.1	7.7	5.5
Wetlands	8.9	18.7	11.4	4.1	6.4	9.3	13.6	5.5	8.4	3.6	8.8	8.1	2.5	21.2
Open water	2.7	7.4	3.8	2.1	4.0	3.6	2.0	1.4	2.6	0.8	2.1	4.8	0.9	2.0
Grass/shrub	1.6	1.3	1.9	1.1	1.4	2.4	1.8	3.1	1.6	2.2	1.8	1.4	1.0	2.2

*Note*: This table provides a land cover analysis for each of the 12 climate assessment regions. Data in the table are adapted from the 2019 National Land Cover Database (NLCD). For the purposes of this broad overview, some land cover categories in the database were combined as follows: open water (NLCD category 11, which includes rivers as well), developed (categories 21 + 22 + 23 + 24), grass/shrub (categories 31 + 52 + 71), forest (categories 41 + 42 + 43), agriculture (categories 81 + 82), and wetland (categories 90 + 95). The data do not include the surface area of the Great Lakes and the marine and coastal district waters that lie within New York State.

^a^ Land cover data were compiled for 10 distinct areas of Indigenous land, including lands under the jurisdiction of eight federally recognized Indigenous Nations in New York State, as well as the Poospatuck Reservation of the Unkechaug Band, which is recognized by the state.

*Source*: Dewitz and US Geological Survey (2021).^12^

Some key features of the state's geography include:

- More than 7600 freshwater lakes, ponds, and reservoirs.^13^
- Abundant rivers and streams, with the St. Lawrence, Mohawk, Susquehanna, Delaware, and Hudson rivers among the largest in the state.
- Atlantic Ocean shoreline along the coast of Long Island and portions of New York City, along with associated estuaries where fresh water mixes with Atlantic salt water (i.e., the Peconic Estuary, the Long Island South Shore Estuary, New York/New Jersey Harbor, Long Island Sound, and the Hudson River Estuary).^14^
- Approximately 850 miles of Great Lakes shoreline, which includes shorelines of lakes Erie and Ontario and the Niagara and St. Lawrence rivers.^15^
- Mountainous areas, including the Adirondack and Catskill ranges, as well as other upland regions such as the Hudson Highlands, the Taconic Range, and portions of the Allegheny Plateau.
- Urban areas, including New York City (the nation's most populous city) and adjacent metropolitan counties, along with Buffalo, Rochester, Syracuse, Albany, and numerous other cities.
- Nearly 7 million acres of farmland.^16^

### 4.2 Government structure

Understanding the structure and authorities of state and local governments in New York State is important for recognizing where decisions about climate policy, adaptation, and resilience are made.

State‐level government includes three branches: legislative, executive, and judicial. The legislative branch consists of the New York State Senate and the New York State Assembly. The Senate and Assembly collaborate to amend and draft New York State laws, subject to the Governor's signature. The executive branch includes four elected offices: the Governor, the Lieutenant Governor, the State Comptroller, and the Attorney General. The executive branch oversees 20 departments—including, for example, Agriculture and Markets, Labor, Education, Health, and Environmental Conservation.^17^ The judicial branch includes a range of courts. In addition to the three branches of state government, New York State has numerous public authorities, which are public benefit corporations authorized by the legislature, with varying levels of autonomy. Public authorities operate some of the state's critical infrastructure, including some roads and bridges, portions of the electric grid, and some mass transit systems.

At the local level, the state is divided into 62 counties, which are further divided into municipalities, including cities, towns, and villages. Counties and municipalities often oversee programs and services that play a role in climate adaptation. For instance, municipalities lead infrastructure improvements and maintenance, social and environmental services, land planning and development, and public health and safety programs. As a result, municipal governments are at the front line for climate action. New York is a home rule state, which means that the state's constitution grants municipalities the right to pass their own laws and regulations on many issues, based on their local needs.

There are nine state‐recognized Tribes in New York, eight of which are also federally recognized, as described in Section 4.5.2. These Tribes are sovereign nations with their own government structures.

### 4.3 Economic landscape

New York State is an economic center, domestically and internationally. As illustrated in Figure 1-5, the largest economic sectors in the state's $1.9 trillion^18^ economy are finance, insurance, real estate, rental, and leasing, which together accounted for more than a third of the state's gross domestic product (GDP) in 2021.^19^ In 2020, there were approximately 9.4 million employed workers.^20^ Health care and social assistance (1.75 million jobs) represented the largest employment sector, followed by local and state government (1.29 million jobs).^21^ Small businesses are also a key provider of employment. As of 2020, 98% of businesses in New York State reported having fewer than 100 employees, with small businesses accounting for over 53% of total employment.^22^

**FIGURE 1-5:**
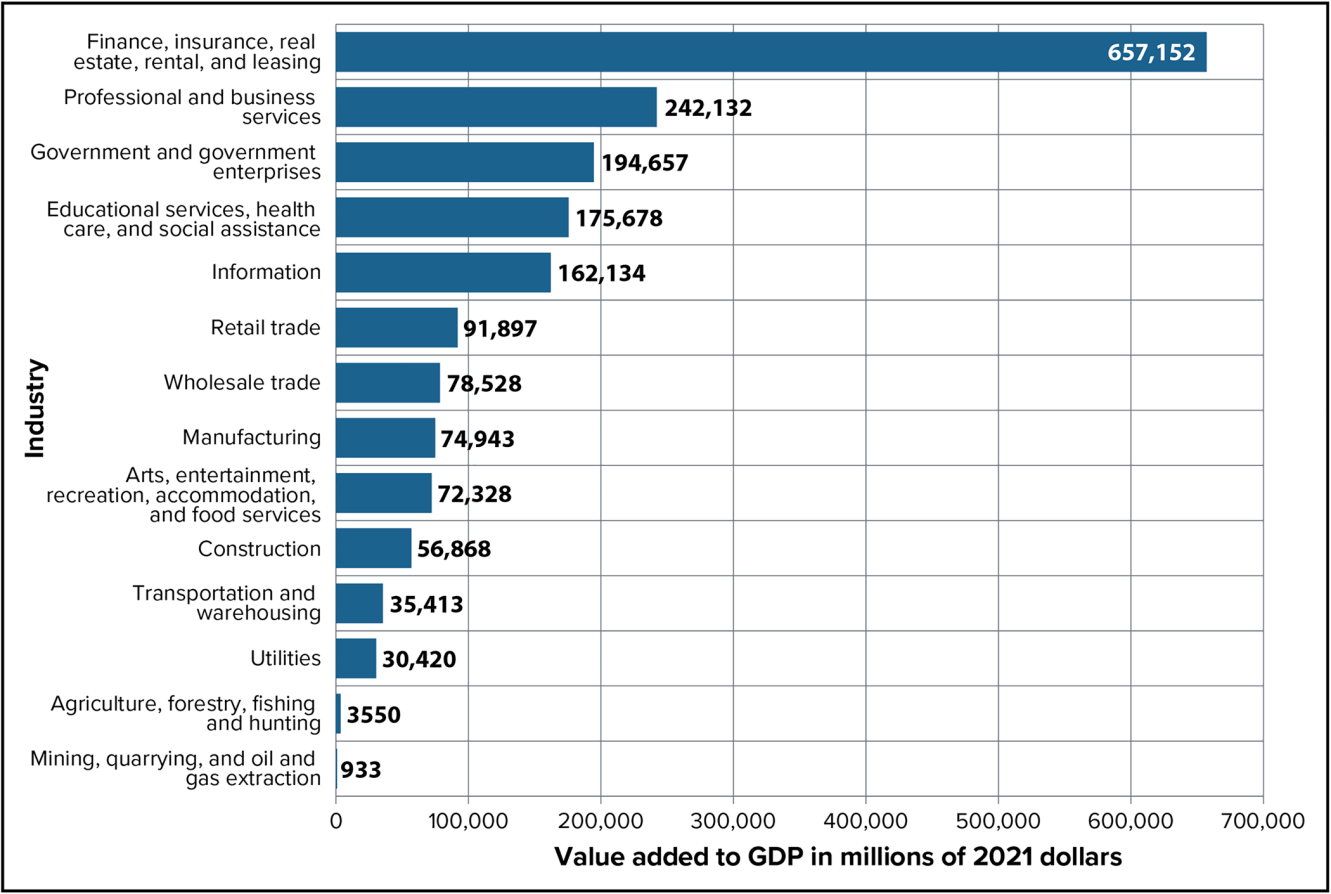
Value added to gross domestic product (GDP) in New York State, 2021, by industry (in millions of current US dollars, not adjusted for inflation). *Source*: Data from US Bureau of Economic Analysis (n.d.).^19^

On the surface, New York State's economy is strong compared with the rest of the country, based on GDP, employment, and wage levels. However, a closer look reveals two regional economies—one substantially larger and stronger than the other (Table 1-2). The per capita income in the state overall is close to $69,500 per year, well above the national average of about $59,000,^23^ due in part to New York City's high wages in the financial and insurance sectors. However, when New York City and surrounding metropolitan areas are excluded, the per capita income for the rest of the state falls to approximately $54,400 per year, which is below the national average. Weekly wages in New York County (Manhattan) averaged approximately $2500 in the third quarter of 2021, whereas the average weekly wage in many rural counties was less than $1000.^23^

**TABLE 1-2:** Regional differences in gross domestic product (GDP) and income, 2020.

	New York City, Long Island, Westchester, Orange, and Rockland counties	Rest of New York State	New York State overall	United States overall
Per capita GDP^a^	$103,058	$56,255	$87,463	$64,332
Per capita income^b^	$77,096	$54,454	$69,552	$59,164
Population^c^	13,469,980	6,731,269	20,201,249	331,449,281

^a^ 2020 per capita GDP from US Bureau of Economic Analysis (n.d.)^26^ and US Census Bureau (2020).^27^ Values calculated by summing GDP for selected counties and dividing by total population in those counties.

^b^ 2020 per capita income from US Bureau of Economic Analysis (2023)^28^, ^29^ and US Census Bureau (2020).^27^ Values calculated by summing personal income for selected counties and dividing by total population in those counties.

^c^ 2020 population from US Census Bureau (2020).^27^

Additionally, income inequality is prevalent throughout New York State. According to the 2019 Gini Index—a measure of income inequality—New York has the highest wealth disparity of any state in the country.^24^ Differences in income can be observed across racial and ethnic groups. For instance, for the 2018–2020 period, the median annual income of all households was roughly $75,000.^25^ During that same period, the median annual income of non‐Hispanic white households was roughly $30,000 higher than that of Hispanic households and non‐Hispanic Black households.^25^

### 4.4 Demographic landscape

New York is one of the most demographically diverse states in the country. According to the 2020 US Census, the state has a population of 20.2 million,^30^ with more than 40% residing in New York City.^31^ Close to 45% of the state's population self‐identifies as Black/African American; American Indian or Alaska Native; Asian; Native Hawaiian or other Pacific Islander; some other race; or two or more races. Roughly 20% of the population identifies their ethnicity as Hispanic or Latino.^30^

New York's cultural richness has grown from the state's history as a major entry point into the United States.^32^ As of 2020, people born outside of the country represented nearly a quarter of the state's residents and more than a 10th of its workforce.^20^, ^33^ Domestic migration also affects population and settlement patterns. New York City recently experienced net population loss as residents, largely from affluent neighborhoods and communities with higher population densities, moved to surrounding counties and to other states.^34^ Other areas of New York State—such as the Adirondacks, the Champlain Valley, the Great Lakes, and the Central/Finger Lakes region—experience seasonal migration during the summer months. The Adirondacks, for example, are home to approximately 130,000 year‐round residents and an additional 200,000 seasonal residents.^35^

Nearly 17% of New York's residents are 65 years old or older^31^—the fastest growing age group in the state.^36^ Approximately 21% of the population is under the age of 18, and 6% of the population is under the age of 5.^31^

### 4.5 Vulnerable populations

Although all populations and communities feel the impacts of climate change, some populations may face disproportionate impacts. For many groups, particularly Indigenous Peoples and members of marginalized racial and ethnic groups, climate vulnerabilities are rooted in legacies of dispossession, displacement, discrimination, and redlining.^37^, ^38^, ^39^, ^40^ Across society at large, these vulnerabilities are also influenced by various types of discrimination, injustice, and inequity that continue in some form to this day.^41^, ^42^, ^43^ For example, communities that are disproportionately impacted by environmental pollution may be especially vulnerable to climate change impacts. Likewise, older adults in low‐income communities may be experiencing housing, energy, food, and health insecurities, all of which could be exacerbated by climate change. Understanding existing inequities is essential for understanding vulnerabilities to climate change.

#### 4.5.1 Discriminatory practices in housing

Historical injustices such as redlining, urban renewal, and exclusionary zoning have profound implications for millions of New Yorkers. In many places across the state, discriminatory practices from as much as a century ago continue to contribute to inequitable vulnerability to climate impacts.

##### 4.5.1.1 Redlining

Redlining is foundational to the housing patterns observed today in many cities throughout the United States, where housing is segregated along lines of race and nationality, income, and environmental quality. Redlining practices were implemented by the federal Home Owners’ Loan Corporation (HOLC), created in 1933 to help systematically prioritize investment in the built environment. During the Great Depression, when many homeowners across the country faced foreclosure due to property depreciation and falling household incomes, the HOLC granted long‐term mortgage loans to around 1 million homeowners nationwide. Mortgage loans were allocated based on maps in which neighborhoods were color‐coded according to community characteristics that influenced perceived investment desirability. Areas colored red were often inhabited by Black and immigrant communities and annotated with discriminatory descriptions. These areas were deemed undesirable for investment and ineligible for federally backed loans.

Although the HOLC ceased issuing its own loans in 1936,^44^ the organization and its maps persisted, and redlining practices remained legal through 1968. The impacts of redlining—along with other discriminatory practices, such as those employed through the Federal Urban Renewal Program—contributed to residential segregation patterns, homeownership disparities, and deteriorating infrastructure (resulting in lower property values) that are still present to this day.^39^ Furthermore, in many redlined neighborhoods, these practices led to conditions that have exacerbated climate impacts. Box 3 provides an example of how historical redlining in Yonkers, New York, has contributed to inequitable exposures to extreme heat.

BOX 3 **Redlining and inequitable heat exposure**

In partnership with Climate Safe Neighborhoods, Groundwork Hudson Valley completed an in‐depth analysis of the relationship between historical redlining and climate impacts in Yonkers, New York.^45^ Their analysis examined the locations of redlined areas on 1930s HOLC zoning maps and compared those with modern day tree coverage, impervious surface coverage, and temperature data.

Higher temperatures are often observed in urban neighborhoods with large areas of impervious surfaces, like asphalt and buildings, and with little natural land cover, like trees. This phenomenon is known as the urban heat island effect.^46^ As illustrated in Figure 1-6, temperatures within an urban environment can vary drastically between neighborhoods, depending on the presence or absence of certain built or natural features.^47^ Specifically, the map shows that surface temperatures in previously redlined neighborhoods are, on average, nearly 3°F hotter than those in areas highly rated by the HOLC, outlined in blue and green. The higher temperatures are directly related to lower tree canopy coverage and higher amounts of impervious surface coverage in previously redlined areas, compared with non‐redlined areas. These factors combined result in exposure to extreme heat and a disproportionate impact to redlined communities. Extreme heat exposure can have negative impacts that include increased heat‐related illness and mortality, increased energy costs, and impaired air quality.^48^

The experience in Yonkers is not unique; many communities have a similar pattern of higher temperatures in redlined neighborhoods.^48^ The disproportionate impact of extreme heat on redlined communities is only one example of how past policies, even when short‐lived, can have lasting impacts on communities.

**FIGURE 1-6:**
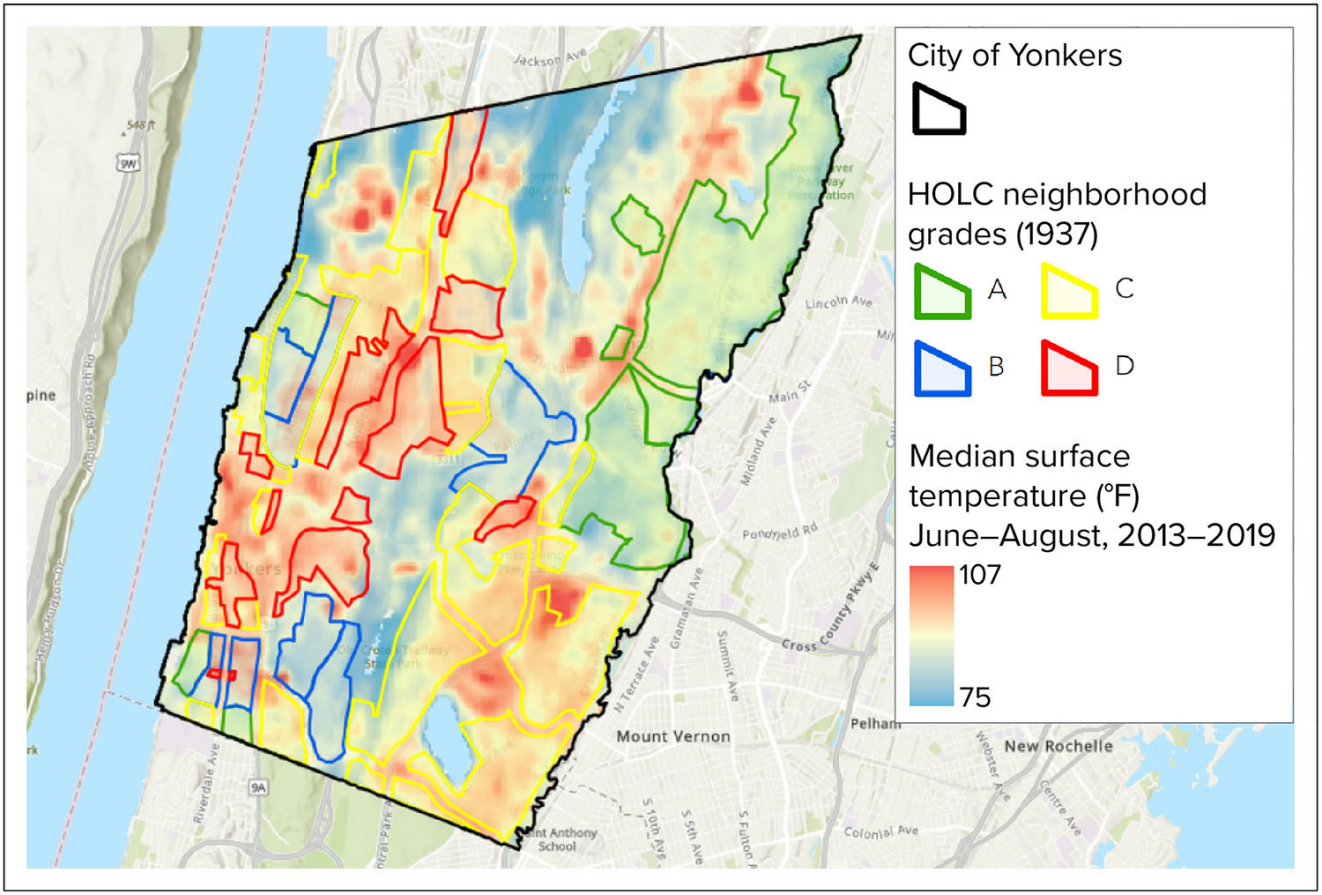
Relationship between median surface temperatures and historic Home Owners’ Loan Corporation (HOLC) neighborhood grades, Yonkers, New York, 2013–2019. *Source*: Figure adapted from Groundwork Hudson Valley (n.d.).^45^ (Map image is the intellectual property of Esri and is used herein under license. Copyright © 2020 Esri and its licensors. All rights reserved.)

##### 4.5.1.2 Urban renewal

Urban renewal efforts that took place from the 1950s to the 1970s also contributed to some of the segregated housing patterns and other disparities that are observed today.^39^ Such efforts were undertaken through the Housing Act of 1949, which provided federal funds for urban redevelopment throughout the country. Title 1 of the Housing Act set aside $1 billion in federal aid to assist localities with the clearing and redeveloping of what the Act referred to as “slums and blighted areas.”^49^ These areas often corresponded to redlined areas, which had been systematically denied investment.^49^ Urban renewal projects often used eminent domain to acquire buildings. The buildings were then razed and the land redeveloped for highways, middle‐class housing, and commercial and office buildings. Communities within New York City experienced displacement from this program, as did people in other cities and towns including Buffalo, Albany, Rochester, Syracuse, and White Plains.^50^ Remaining residents in areas bisected by highways found their communities disconnected and subjected to noise and air pollution. Climate change is now another stress on top of the challenges that these communities already face.

##### 4.5.1.3 Zoning

Zoning decisions made as much as a century ago have indirect yet important implications for environmental justice and climate adaptation today. Zoning helped determine the locations of power plants and industrial facilities and thereby laid the groundwork for communities that would thereafter be affected by locally undesirable land uses and greater pollution burdens. More recently, these communities have begun to face additional risks, such as the threat of contamination during flooding made worse by climate change.

Today's zoning decisions often have similar implications. Across the United States, including in New York State, exclusionary zoning restricts which types of homes can be built in neighborhoods. Some common examples of exclusionary zoning policies include limits on multifamily housing development, building height restrictions, and minimum lot size requirements. These restrictions reduce housing supply, resulting in higher housing costs and fewer housing options.^51^ Such policies have been found to fuel racial and economic segregation, ultimately contributing to economic and health disparities that climate change exacerbates.^52^, ^53^ New York City and its surrounding suburbs serve as an example, with exclusionary zoning contributing to some of the most segregated neighborhoods in the country.^54^ Exclusionary zoning represents an ongoing systemic injustice, excluding low‐income groups from living in areas that are potentially more resilient.

#### 4.5.2 Tribal Nations and Indigenous Peoples

Indigenous communities have resided within what is now known as New York State for at least 13,000 years. The dispossession and displacement of Indigenous Peoples following European settlement in the 17th century resulted in loss of lands, along with a loss of access to vital natural resources and sacred sites—the consequences of which are still felt today. In addition, the siting of polluting industries on and near Indigenous lands has led to the current toxic contamination of culturally important ecosystems. Two well‐known examples are the polluting of Onondaga Lake (a sacred site referred to by the Haudenosaunee [Iroquois] as Where the Water Meets the Willows) and the contamination of the Saint Regis Mohawk Tribe's water supply due to industrial dumping.^55^, ^56^

According to the 2020 Census, New York State is home to almost 400,000 residents with American Indian or Alaska Native heritage, representing 2% of the state's population.^30^ More than one third of these people identify as being solely American Indian or Alaska Native. Additionally, more than 40,000 residents have Native Hawaiian or Other Pacific Islander heritage.^30^ Of the Indigenous population, about 11,000 people, or less than 3%, live within Tribal reservations (Table 1-3). Reservation territory in the state totals approximately 137 square miles (87,650 acres), though the borders of these territorial areas change with land claim legal actions and purchases.

**TABLE 1-3:** Tribal Nation reservations in New York State.

Reservation	Population (2020)	Area (sq miles)	Location (counties)
Allegany (Seneca Nation)	1064	48.8	Cattaraugus
Cattaraugus (Seneca Nation)	2676	34.4	Erie, Cattaraugus, Chautauqua
Oil Springs (Seneca Nation)	20	1.00	Cattaraugus, Allegany
Oneida Nation	9	0.05	Madison
Onondaga Nation	831	9.28	Onondaga
Poospatuck (Unkechaug Nation)	436	0.09	Suffolk
Saint Regis Mohawk (Mohawk Nation)	3663	21.0	Franklin
Shinnecock (Shinnecock Nation)	819	1.29	Suffolk
Tonawanda (Tonawanda Seneca Nation)	261	11.9	Genesee, Erie, Niagara
Tuscarora Nation	1145	9.13	Niagara
Total	10,924	136.9	

*Source*: Data from New York State GIS Resources (n.d.).^57^

The Indigenous population includes citizens of the following nine federally recognized or state‐recognized Tribal Nations: Cayuga Nation, Oneida Indian Nation, Onondaga Nation, Saint Regis Mohawk Tribe, Seneca Nation of Indians, Shinnecock Indian Nation, Tonawanda Seneca Nation, Tuscarora Nation, and Unkechaug Indian Nation (Figure 1-7). Additional Tribal Nations that are no longer located in New York State but are still connected to the region include the Delaware Nation (Oklahoma), Delaware Tribe of Indians (Oklahoma), and Stockbridge‐Munsee Band of the Mohican Nation of Wisconsin. There are also other Indigenous communities that live in New York and surrounding states, such as the Ramapough Lenape and Montaukett.

**FIGURE 1-7:**
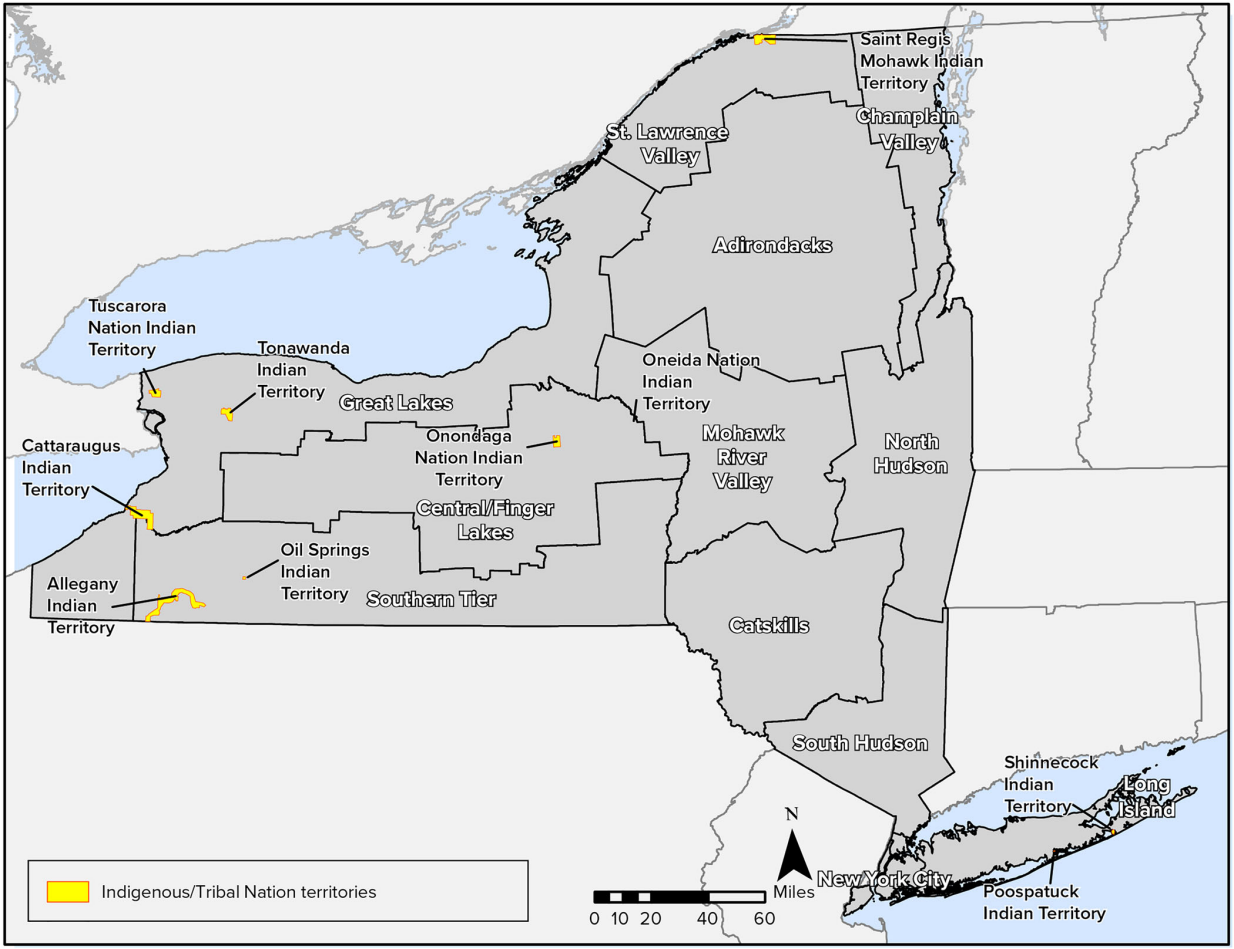
Indigenous Tribes/Nations in New York State. *Source*: Data from New York State GIS Resources (n.d.).^57^

Tribal and Indigenous populations experience socioeconomic vulnerabilities, including relatively high rates of poverty and low levels of educational attainment. These vulnerabilities affect the ability of Indigenous communities to prepare for and respond to climate impacts.^58^, ^59^ Overall, the colonization of large swaths of traditional territories, the dispossession and pollution of agricultural lands and water bodies, and the forced migration of Indigenous Peoples to lower quality lands have contributed, along with present socioeconomic disparities, to Tribal Nations having greater exposure to climate‐related risks.^60^

#### 4.5.3 Rural populations

Rural areas cover nearly 90% of the state and represent nearly half of the more than 1600 municipalities, though only 12% of New Yorkers reside within them.^61^ Many rural communities depend on natural resource–based economic activities such as agriculture, forestry, and tourism. Thus, rural communities not only face considerable risks to their agricultural production and other valued industries due to climate impacts but also face risks to their livelihoods, infrastructure, and quality of life.^62^ These risks also have statewide implications, as rural areas provide much of the food production and many of the ecosystem services that support the state's larger urban areas.^63^

Among counties in New York with 14% or more of the population living in poverty, most are concentrated in rural areas, although Kings County (Brooklyn) and Bronx County in New York City have the highest poverty rates in the state.^64^ Rural communities are susceptible to overlapping social and economic stresses, such as endemic poverty, poor health, and drug dependency, in addition to climate stresses that exacerbate existing vulnerabilities. The population profile of rural areas also adds to their vulnerability, as these areas tend to contain relatively high percentages of older residents, many of whom are living on limited or fixed incomes. The aging and shrinking of populations in rural communities contributes to reduced tax bases, which in turn affect the capacity of these areas to prepare for, respond to, and recover from climate extremes.^65^ Compared with larger metropolitan communities, rural towns tend to have more limited capacities for developing and implementing climate resilience and preparedness plans.

#### 4.5.4 Environmental justice and “disadvantaged” communities

New York State defines environmental justice as “the fair and meaningful treatment of all people, regardless of race, income, national origin or color, with respect to the development, implementation, and enforcement of environmental laws, regulations and policies.”^66^ The principle of environmental justice recognizes that some communities face disproportionate exposure to environmental pollutants associated with industry, transportation, sewage treatment, and other similar activities. Environmental justice concerns are particularly prominent in densely populated communities of color. Communities with environmental justice concerns have often experienced decades of private disinvestment and a lack of meaningful government support. Such communities are more likely than others to have been redlined, designated as blighted, or targeted for slum clearance or urban renewal. Climate‐related stressors can overlap with existing forms of disinvestment and environmental harms.^67^ Compounding these double exposures to climate and social stressors, residents of these communities also often lack financial resources, such as flood insurance, to aid in recovery from extreme events such as floods and coastal storms.

The NYSDEC defines Potential Environmental Justice Areas as US Census block groups of 250–500 households each that, in the census, had populations that met or exceeded at least one of the following statistical thresholds:

- At least 52.42% of the population in an urban area reported themselves to be members of minority groups;
- At least 26.28% of the population in a rural area reported themselves to be members of minority groups;
- At least 22.82% of the population in an urban or rural area had household incomes below the federal poverty level.

Figure 1-8 shows areas of the state that have been designated as Potential Environmental Justice Areas, as well as census tracts that have been designated as “disadvantaged” communities in association with the Climate Act. The Climate Act charged the Climate Justice Working Group with developing criteria for identifying disadvantaged communities based on geographic, public health, environmental hazard, and socioeconomic indicators. Areas with high concentrations of census tracts that meet the criteria are located in both urban and rural areas of the state, including the New York City, South Hudson, Catskills, and Great Lakes assessment regions (Table 1-4).

**FIGURE 1-8:**
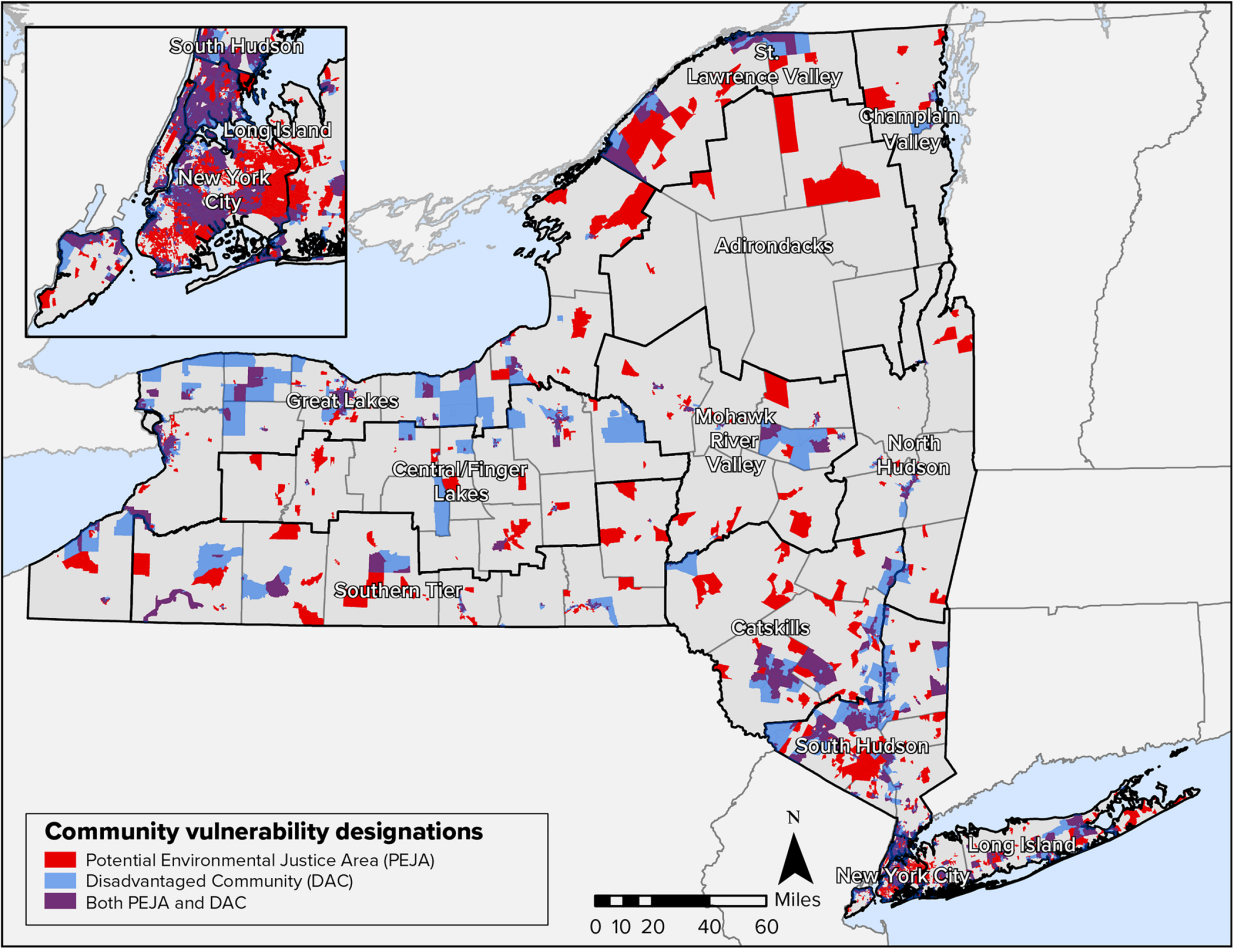
Potential Environmental Justice Areas (PEJAs) and disadvantaged communities (DACs) in New York State, 2023, as identified by the NYSDEC Office of Environmental Justice and the Climate Justice Working Group. *Source*: PEJA data from NYSDEC, Office of Environmental Justice (2022).^68^ DAC data from NYSERDA (2023).^69^

**TABLE 1-4:** Percentage of census tracts within each assessment region designated as disadvantaged communities.

Assessment region	Percentage of census tracts identified as disadvantaged communities (%)
Adirondacks	0
Catskills	38
Central/Finger Lakes	27
Champlain Valley	12
Great Lakes	37
Long Island	14
Mohawk River Valley	26
New York City	44
North Hudson	22
South Hudson	42
Southern Tier	26
St. Lawrence Valley	25

*Source*: Data from NYSERDA (2023).^69^

Elsewhere in this assessment, the terms “overburdened,” “underserved,” “priority,” and “vulnerable” are used in place of “disadvantaged” when not referring directly to the Climate Act designation, as some communities have expressed objections to the term.

#### 4.5.5 Other vulnerable populations

Additional populations and communities that face disproportionate risks from climate impacts include but are not limited to

- **Immigrant populations**. Populations that have newly immigrated to New York State may have heightened vulnerabilities due to factors such as higher‐than‐average rates of poverty, limited English proficiency, undocumented status, and anti‐immigrant discrimination.
- **Unhoused and housing‐insecure populations**. Many individuals experiencing housing insecurity have underlying health problems that can heighten sensitivity to extreme weather events, especially as lack of safe and secure housing exacerbates exposure to climate hazards such as heat waves, heavy rainfall events, and flooding. The vulnerability of unhoused people to extreme heat has been identified as a particular concern in New York City.^70^
- **Incarcerated populations**. Incarcerated individuals are uniquely vulnerable to the impacts of climate change due to a combination of health conditions and environmental factors.^71^, ^72^, ^73^, ^74^, ^75^, ^76^ Although the extent of potential impacts needs further research, important factors include living conditions in prisons (such as a lack of proper temperature control) and limited mobility. In addition, incarcerated individuals often have a high rate of chronic health conditions. As of 2021, approximately 30,000 individuals were in prisons across the state.^77^
- **People with disabilities**. People with disabilities may be particularly vulnerable to climate change impacts, especially those that affect the availability of vital health services, adequate housing, and accessible employment.^78^ The aftermath of Superstorm Sandy in New York City serves as an example of the special vulnerabilities of this population, as many residents with disabilities were essentially trapped on the upper floors of apartment buildings due to power outages and associated lack of elevator service.^79^
- **Children**. Children are physically more vulnerable to the effects of extreme heat, drought, and weather‐related disasters due to their size, physiology, and behavior.^80^ Moreover, children are more susceptible to indirect effects of climate change such as food shortages, conflict, negative economic impacts, and migration.^81^
- **Older adults**. By 2040, 22% of New York State's population is expected to be age 65 or older.^82^, ^83^ Older adults typically have greater sensitivity to climate‐related hazards such as heat and air pollution. In addition, older individuals who have physical mobility challenges or who depend on medical care and assistance for daily life face increased vulnerability during extreme weather events.^84^

The Human Health and Safety and Society and Economy chapters discuss the unique climate vulnerabilities of these groups and others in additional depth.

#### 4.5.6 Equity and justice considerations and opportunities

The Climate Act aims to ensure that all New Yorkers, but especially priority communities, benefit from greenhouse gas emissions reduction and associated economic opportunities and improvements in air quality as the state transitions to renewable energy.^85^ Centering equity in adaptation and resilience initiatives is equally important for a successful and sustainable climate change response. Within environmental and climate justice literature, several forms of equity are commonly cited.^67^, ^86^, ^87^, ^88^ Awareness of these components of equity is critical for developing inclusive and durable climate change responses:^67^

- **Distributional equity** concerns uneven capacities to adapt across different communities and groups, as well as inequalities in the benefits and harms of adaptation actions.^89^
- **Procedural equity** focuses on the representation and inclusion of affected groups and communities in adaptation processes, decision‐making, and resilience actions.^67^
- **Contextual equity** emphasizes underlying factors including preexisting social and economic inequalities, historical legacies of land dispossession, and racial and ethnic discrimination and related injustices that create an “uneven playing field” for particular communities.^67^, ^86^

Climate justice efforts are often focused on the distributional effects of climate change because these effects disproportionately burden low‐income communities and communities of color.^90^ Each sector covered in this assessment and every community in New York State has the potential to contribute to climate solutions that reduce vulnerabilities, enhance resilience, and foster just transitions.

The Climate Act addresses concerns about distributional, procedural, and contextual equity for “disadvantaged” communities. Specifically:

- It addresses distributional equity by requiring that disadvantaged communities receive at least 35% (with a goal of 40%) of the benefits from spending and investments in clean energy and energy efficiency programs. This goal also extends to energy‐related investments in housing, workforce development, pollution reduction, transportation, economic development, and low‐ and moderate‐income energy assistance.^91^
- It addresses procedural equity by requiring the formation of the Climate Justice Working Group—made up of representatives from environmental justice communities across the state as well as from select state agencies—to advise on the draft scoping plan and define the criteria used to identify disadvantaged communities.
- It employs principles of contextual equity by acknowledging, in its language, the heightened vulnerability of communities that “bear environmental and socioeconomic burdens as well as legacies of racial and ethnic discrimination” and by taking concrete steps to prioritize the health and safety of those communities.

Another key equity and justice consideration is ensuring that climate change responses are inclusive and sustainable at the local level.^67^, ^92^, ^93^, ^94^, ^95^ To achieve this result, local communities must be involved throughout the planning and decision‐making process. Inclusivity and sustainability also require just allocation of resources (e.g., climate funding and data) and awareness of both long‐standing inequitable environmental practices and more recent climate justice discussions.^93^, ^96^, ^97^, ^98^ The “Data justice” and “Language justice” text boxes provide examples.

BOX 4 **Data justice**

*For just decision‐making and action to occur, people need to be able to access and shape environmental data specific to their communities*.

In *Investing in Data Capacity for Community Change*, Hendey et al.^99^ define a community with data capacity as “one where people can access and use data to inform efforts to understand and improve outcomes where they live.” They find that even when data capacity does exist in a community, it is not held equitably across groups of people, organizations, and institutions. As a result, there is potential for well‐intentioned efforts to contribute to disparity and harm. Data access and support are fundamental to data capacity.^99^ A related concept is that of literacy in the age of data where data inclusion is framed as a critical component of social inclusion.^100^

As large data sets and associated tools are increasingly used to better understand, prepare for, and address climate change impacts, intentional consideration of context and process is necessary to avoid unintended consequences for the most vulnerable populations. Data collection, aggregation, analysis, interpretation, dissemination, and access are all important components of a climate data justice framework to advance equitable community climate adaptation.^101^

Johnson^102^ advocates for “information justice,” arguing that social privilege can create serious inequities in who can access and use data. This idea complements the concept of data inclusion noted above and could be further advanced by the benefits of co‐designing and co‐performing data collection and research activities with impacted communities (and providing funding to support these activities).

Similarly, the Environmental Data and Governance Initiative (EDGI), first formed in response to the 2016 US elections and ensuing public concern about the future integrity of US environmental agencies and policy, is considering infrastructures required for community stewardship of data.^103^ Drawing from environmental justice, geospatial, and other data studies, EDGI is developing an environmental data justice framework, which emphasizes community‐based environmental data collection, public accessibility of environmental data, and environmental data platforms supported by an open‐source online infrastructure that can be used and modified by local communities.^104^ Moreover, the draft framework advocates for using community data to envision alternative futures, rather than using them to characterize communities by a general condition that can prove to be stigmatizing. The framework also highlights how lack of environmental data collection can, in itself, represent injustice.^103^

In summary, data justice includes careful consideration of what is considered “data” (who controls and collects it, who defines what is important), as well as consideration of data accessibility (encompassing literacy and capacity concepts).

BOX 5 **Language justice**

*Communicating climate and health information to the public in multiple languages is essential but often overlooked*.

Risk communication mechanisms related to climate threats are not always constructed to deliver warnings in multiple languages, and many state and local public health departments across the country are not delivering messages about the health ramifications of climate change.^105^ Innovative approaches to multilingual communication, including efforts to improve climate change and health awareness in younger populations within digital channels, can help to broaden public awareness. Public health departments and other health communication professionals should also consider the inclusion of Indigenous languages and/or inclusion of braille; the use of large‐print formats for publications; accessible web design; and American Sign Language interpretation. In March 2022, following the fatalities inflicted by Hurricane Ida primarily on individuals of Asian descent with limited proficiency in English or Spanish, New York's Attorney General called on the US Secretary of Commerce and the Acting Director of the National Weather Service to expand the language accessibility of severe weather alerts to more effectively reach all immigrant communities.^106^

## AUTHOR CONTRIBUTIONS

C.G.: Drafting; compiling; and revising the manuscript. S.A.: Drafting and revising the manuscript; coordinating demographic data analysis. D.C.: Drafting the manuscript.

## COMPETING INTERESTS

The authors declare no competing interests.

### PEER REVIEW

The peer review history for this article is available at: https://publons.com/publon/10.1111/nyas.15202
